# Maternal gastrointestinal nematode infection alters hippocampal neuroimmunity, promotes synaptic plasticity, and improves resistance to direct infection in offspring

**DOI:** 10.1038/s41598-024-60865-2

**Published:** 2024-05-10

**Authors:** Sophia C. Noel, Jeanne F. Madranges, Jean-David M. Gothié, Jessica Ewald, Austen J. Milnerwood, Timothy E. Kennedy, Marilyn E. Scott

**Affiliations:** 1 Department of Neurology and Neurosurgery, Montreal Neurological Institute-Hospital, 3801 University Street, Montreal, QC H3A 2B4 Canada; 2 Institute of Parasitology, McGill University (Macdonald Campus), 21,111 Lakeshore Road, Sainte-Anne de Bellevue, QC H9X 3V9 Canada

**Keywords:** Long-term potentiation, Learning and memory, Neuroimmunology, Parasitic infection

## Abstract

The developing brain is vulnerable to maternal bacterial and viral infections which induce strong inflammatory responses in the mother that are mimicked in the offspring brain, resulting in irreversible neurodevelopmental defects, and associated cognitive and behavioural impairments. In contrast, infection during pregnancy and lactation with the immunoregulatory murine intestinal nematode, *Heligmosomoides bakeri*, upregulates expression of genes associated with long-term potentiation (LTP) of synaptic networks in the brain of neonatal uninfected offspring, and enhances spatial memory in uninfected juvenile offspring. As the hippocampus is involved in spatial navigation and sensitive to immune events during development, here we assessed hippocampal gene expression, LTP, and neuroimmunity in 3-week-old uninfected offspring born to *H. bakeri* infected mothers. Further, as maternal immunity shapes the developing immune system, we assessed the impact of maternal *H. bakeri* infection on the ability of offspring to resist direct infection. In response to maternal infection, we found an enhanced propensity to induce LTP at Schaffer collateral synapses, consistent with RNA-seq data indicating accelerated development of glutamatergic synapses in uninfected offspring, relative to those from uninfected mothers. Hippocampal RNA-seq analysis of offspring of infected mothers revealed increased expression of genes associated with neurogenesis, gliogenesis, and myelination. Furthermore, maternal infection improved resistance to direct infection of *H. bakeri* in offspring, correlated with transfer of parasite-specific IgG1 to their serum. Hippocampal immunohistochemistry and gene expression suggest Th2/Treg biased neuroimmunity in offspring, recapitulating peripheral immunoregulation of *H. bakeri* infected mothers. These findings indicate maternal *H. bakeri* infection during pregnancy and lactation alters peripheral and neural immunity in uninfected offspring, in a manner that accelerates neural maturation to promote hippocampal LTP, and upregulates the expression of genes associated with neurogenesis, gliogenesis, and myelination.

## Introduction

Prenatal exposure to viral and bacterial pathogens has been identified as a risk factor for neurodevelopmental disorders, including autism spectrum disorder (ASD) and schizophrenia^1,2^. Rodent studies have shown that prenatal exposure to these pathogens activates microglia- and astrocyte-mediated neuroinflammation which impairs neuron and oligodendrocyte development and survival, reduces myelination, disrupts synaptic plasticity, and results in irreversible neurodevelopmental defects, impaired cognitive function, and abnormal behaviors^3^. Administration of anti-inflammatory agents to infected mothers during pregnancy and lactation has been effective in dampening neuroinflammation in offspring and preventing the emergence of neurodevelopmental defects and associated behaviors^4,5^.

Gastrointestinal (GI) helminths are ubiquitous in mammalian populations and survive in the host by releasing a variety of immunoregulatory factors that allow them to evade the immune system^6,7^. Via their immunoregulatory abilities, GI helminths dampen inflammatory and pathologic processes and prevent or ameliorate a number of hyper-immune diseases; including, allergy, autoimmune, and inflammation-associated neurological diseases^6–8^. It has been proposed that prenatal exposure to GI helminths alters offspring immunity^8–10^, which may have life-long consequences for their brain function and behavior^8^.

Our lab has focused on the influence of maternal GI nematode infection during pregnancy and lactation upon neurodevelopment and cognitive function in offspring, using the murine laboratory model *Heligmosomoides bakeri* (also known as *Heligmosomoides polygyrus)*, a strictly intestinal nematode with a direct lifecycle^11^. Maternal *H. bakeri* infection allowed 17 day-old offspring to retain object location memories for 3 h and 3 week-old offspring to retain long-term spatial reference memories for 7 days, in contrast to offspring of uninfected dams who could not retain these memories^12^. As rodents are typically not capable of retaining object location memories for 2 + hours until postnatal day (PD) 24, nor can they retain long-term spatial reference memories for 7 days until PD 60^13,14^, these findings suggested that the maturational processes needed to recall these spatial memories were accelerated in response to maternal *H. bakeri* infection^12^. We also reported that maternal *H. bakeri* infection increased the expression of genes associated with long-term potentiation (LTP) in the brain of seven day old uninfected neonates^15^, raising the possibility that this could underlie the enhanced spatial memory^12^.

LTP of glutamatergic synapses is a form of activity-dependent synaptic plasticity and a leading candidate for the neural substrate underlying learning and memory^16,17^. Spatial memory in rodents is widely regarded as dependent on hippocampal synaptic plasticity^18,19^. Induction of LTP by high frequency stimulation of hippocampal Schaffer collateral (CA3-CA1) synapses requires concerted activation of amino-3-hydroxy-5-methyl-4-isoxazole-propionic acid (AMPA)- and *N*-methyl-d-aspartate (NMDA)-type glutamate receptors^16,17^. NMDARs act as pre- and postsynaptic coincidence detectors that gate LTP induction in response to repeated glutamate synapse activity. Initial AMPAR activity in response to glutamate release depolarizes the postsynaptic cell, facilitating NMDAR-dependent calcium influx upon subsequent glutamate release during high-frequency stimulation (HFS)^16,17^. Calcium influx triggers post-synaptic biochemical cascades, through activation of calcium/calmodulin-dependent protein kinase II (CaMKII), protein kinase C (PKC), and mitogen-activated protein kinases (MAPK), that results in the phosphorylation and insertion of additional AMPARs into the post-synaptic membrane, thus strengthening the synaptic response to future glutamate release^20–22^. LTP in the mouse hippocampal slice preparation is found in the second postnatal week^23^; however depending on strain, this can be delayed until 4- to 5-weeks^24^. Neuroinflammation inhibits LTP, whereas several immunoregulatory factors can promote LTP^25^; as *H. bakeri* induces an immunoregulatory response, we hypothesized that maternal *H. bakeri* infection will induce an immunoregulatory response in the offspring hippocampus, promoting hippocampal LTP to explain the earlier development of spatial memory in the offspring of infected dams^12^.

Immunity against *H. bakeri* relies on a strong T helper type 2 (Th2) immune response involving CD4 + Th2 cells, elevated interleukin (IL)-4, IL-5, IL-9, IL-10, and IL-13 cytokine secretion, high serum levels of IgE and IgG1 antibodies, and activation of alternatively activated macrophages, eosinophilia, and mastocytosis in intestinal tissue^11^. Adult worms, however, induce an immunoregulatory response which aids their long-term survival and involves proliferation of Foxp3 + CD4 + regulatory T (Tregs) cells, tolerogenic dendritic cells, and the potent immunoregulatory cytokines IL-10 and transforming growth factor-β (TGF-β)^11^. Maternal transfer of immunity both in utero and via nursing shapes the developing immune system^10^. Consistent with this, maternal infection with GI nematodes can result in the transfer (via nursing) of maternally derived parasite-specific antibodies^26^ and cells^9^, which alters offspring immunity and protects them from direct infection. We reported that immune stimuli from the *H. bakeri* infected mother may also reach the brain of uninfected offspring, as Th2/Treg pathways were up-regulated in neonatal brains^15,27^. If the neuroimmune system is altered by maternal infection, glial cells (microglia, astrocytes and oligodendrocytes), which have vital roles in brain development and function, and are particularly sensitive to immune stimuli, are likely to be altered.

Microglia regulate neurogenesis, neuronal survival, and participate in synaptic pruning and maturation and are the resident immune cells of the central nervous system (CNS)^28,29^. They are highly plastic and take on a wide repertoire of states and functions depending on immune stimuli. In response to Th1/Th17 cytokines, microglia typically release inflammatory mediators, which if prolonged, can drive neuroinflammation and neurotoxicity^30^. Conversely, in response to Th2/Treg cytokines, microglia are associated with immune regulation, neuroprotection and tissue repair^30,31^. Astrocytes also respond to immune stimuli and are involved in the maintenance and regulation of neuronal function, synaptogenesis, neurotransmitter cycling, metabolic support of neurons, modulation of synaptic transmission and maintenance of the blood–brain barrier^29^. Oligodendrocytes generate and maintain myelin sheaths around axons, necessary for the rapid saltatory propagation of action potentials^32^, with developmental myelination peaking during postnatal week three in mice^33^. Due to the vital roles of glia during neurodevelopment, alterations to their development or function in response to immune stimuli transferred from the *H. bakeri* infected mother may provide a mechanism underlying the behavioural changes previously observed^12^.

We aimed to elucidate the mechanisms responsible for the enhanced spatial memory in the uninfected offspring of *H. bakeri* infected mothers^12^. Specifically, in response to this maternal infection, we found changes to LTP and gene expression in the uninfected offspring hippocampus that are consistent with improved performance in spatial memory. Coincident with these changes, we identified increased density of hippocampal microglia and astrocytes, a higher percentage of CD206 positive microglia, and increased expression of the TGF- β signaling pathway. We provide evidence that maternal GI nematode infection improves the resistance of juvenile offspring to direct infection, shifts the peripheral and neural immune response toward a Th2/Treg phenotype, promotes development of hippocampal LTP and upregulates genetic markers of neurogenesis, gliogenesis and myelination, all consistent with earlier development of spatial memory.

## Results

Here we assessed the influence of maternal *H. bakeri* infection on hippocampal gene expression, the capacity to induce LTP, the neuroimmune system, and resistance to direct infection in offspring. Outbred CD-1 mice were repeatedly infected (or sham-infected) during pregnancy and lactation. Juvenile offspring in litters from 18 uninfected and 20 *H. bakeri* infected dams were used. Mortality was consistently zero in this infection model, as expected^12^.

### Maternal *H. bakeri* infection did not influence dam weight or litter size but lowered offspring weight and length

*Heligmosomoides bakeri* did not significantly affect maternal body weight at gestation day (GD 7), 12 or 17 (all p values > 0.9, Supplementary Fig. 1) or litter size (uninfected: 12.6 ± 0.3 vs. infected: 11.8 ± 0.4; t = 1.66, df = 36, p = 0.11). As is typical of a maternal *H. bakeri* infection^12^, pups born to infected dams had shorter length and lower mass than pups of uninfected dams at PD 20 (all p values < 0.0001, Supplementary Fig. 2).

### Maternal *H. bakeri* infection altered hippocampal gene expression in uninfected offspring

To investigate the mechanisms that underlie the previously reported enhancement of spatial memory^12^*,* we assessed changes in the gene expression profile in the hippocampus of PD 23 pups in response to maternal *H. bakeri* infection. After filtering and normalization of high throughput RNA-seq data, we identified 16,143 genes for differential gene expression analysis. Principal component analysis (PCA) showed no pattern with respect to sex, and differential expression analysis (DEA) of males vs. females yielded only 10 differentially expressed genes (DEGs). Offspring sex was therefore excluded as a variable from the analysis.

**Figure 1:**
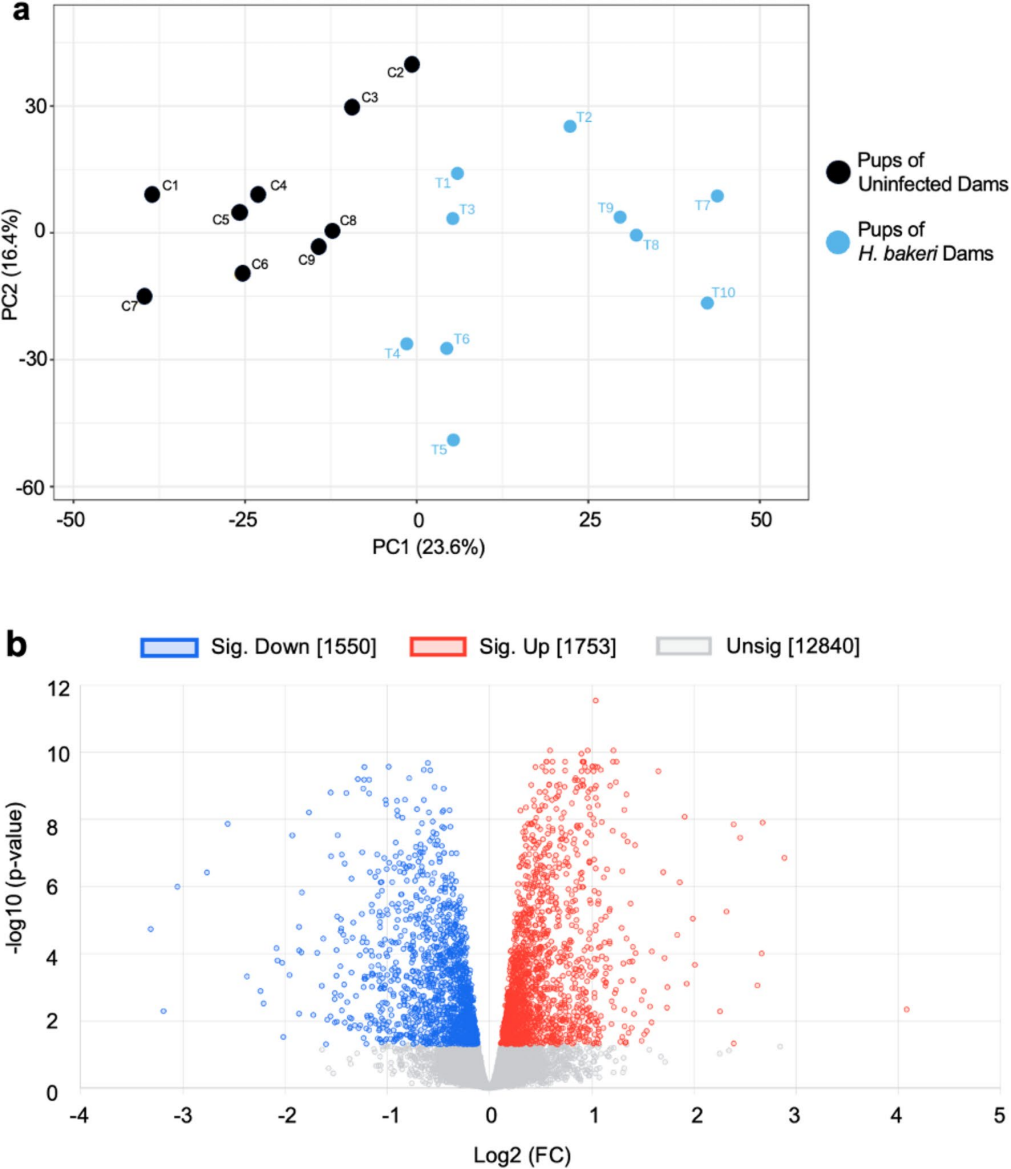
Maternal *H. bakeri* infection influenced offspring hippocampal gene expression. (**a**) Principal component analysis (PCA) showing the relationship between the filtered and normalized gene expression profiles of hippocampal samples from offspring born to *H. bakeri* infected dams (blue) and uninfected dams (black). (**b**) Volcano plot indicating 2166 up-regulated (red, adjusted p-value < 0.05, FDR method) and 2171 down-regulated (blue, adjusted p-value < 0.05, FDR method) genes in the hippocampus of offspring in response to maternal *H. bakeri* infection. Genes with an adjusted p-value > 0.05 are in grey. The x-axis is log2FC and the y-axis is − log10(p-value). n = 9–10 offspring per maternal treatment condition.

**Figure 2:**
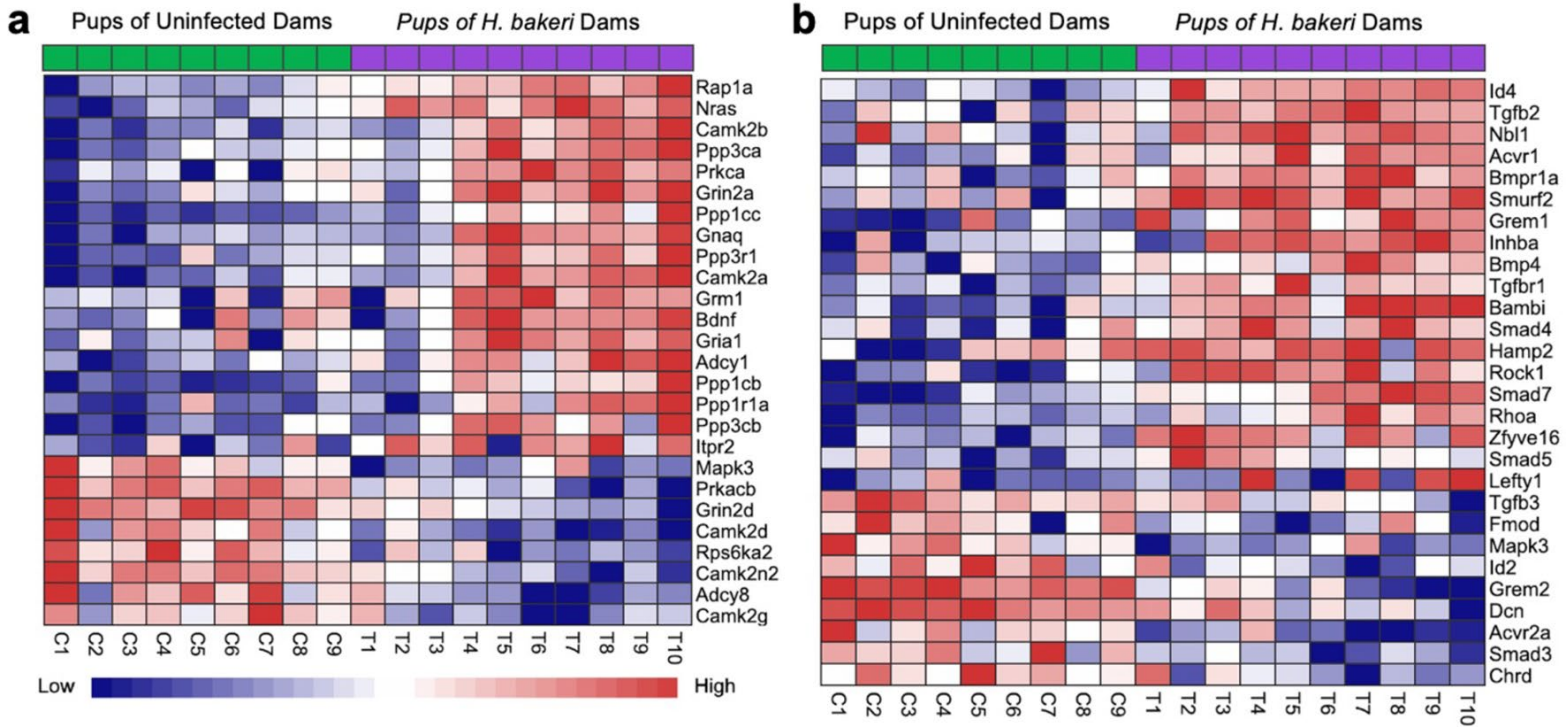
Maternal *H. bakeri* infection increased the expression levels of long-term potentiation and TGF-β signaling KEGG pathways in offspring hippocampus. Heat map of up- and down-regulated genes involved in (**a**) long-term potentiation KEGG pathway (FDR = 0.02) and (**b**) TGF-β signaling KEGG pathway (FDR = 0.03). Blue and red cells correspond to lower and higher expression levels, respectively. n = 9–10 offspring per maternal treatment condition.

**Figure 3:**
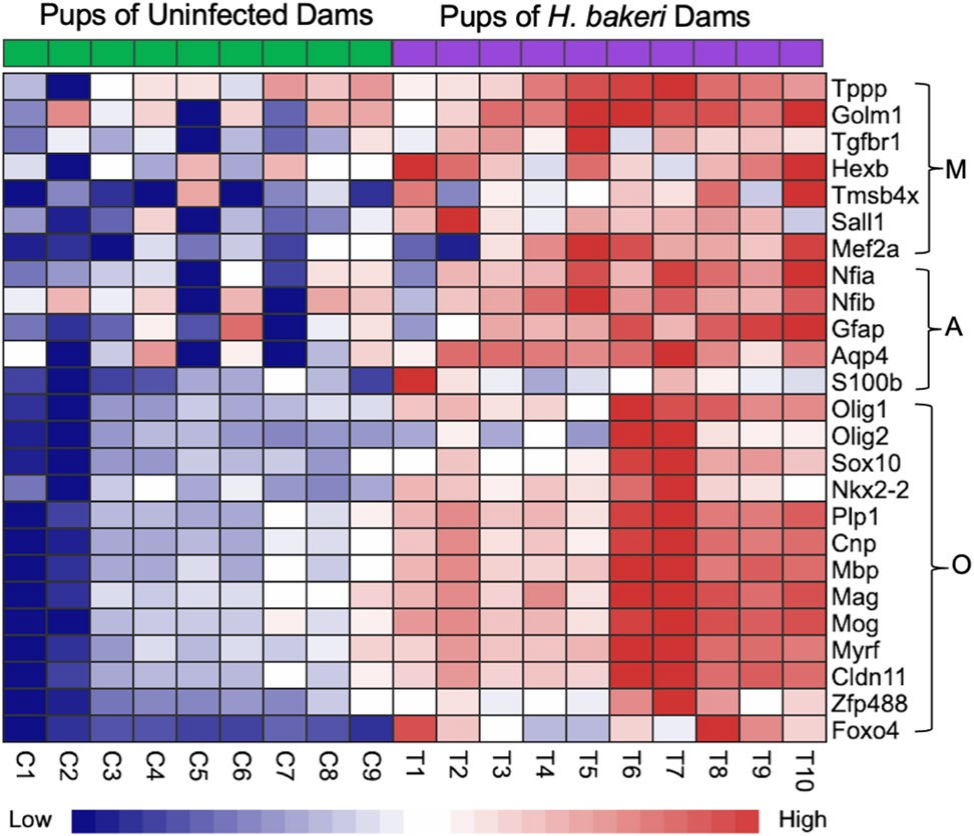
Maternal *H. bakeri* infection increased the expression of genes associated with microglia (M), astrocyte (A) and oligodendrocyte (O) markers in offspring hippocampus. n = 9–10 offspring per maternal treatment condition.

With respect to maternal infection, PCA showed DEGs in two clear clusters, based on treatment, indicating a strong influence of maternal infection on offspring hippocampal gene expression (Fig. 1a). DEA identified 1753 up-regulated, and 1550 down-regulated genes in the pups of *H. bakeri* infected dams (Fig. 1b, Supplementary Table 1). Hypergeometric tests identified 38 KEGG pathways and 67 GO BP terms that were overrepresented in the list of DEGs (FDR < 0.05; Supplementary Table 2). Of particular interest, the LTP (FDR = 0.02; Fig. 2a), glutamatergic synapse (FDR = 0.001; Supplementary Table 3), MAPK signaling (FDR = 0.009; Supplementary Table 4), neurogenesis (FDR = 1.26E-05; Supplementary Table 5), gliogenesis (FDR = 0.0001; Supplementary Table 6), and TGF-β signalling (FDR = 0.03; Fig. 2b) pathways had higher expression levels in offspring from infected mothers. A number of markers associated with microglia (*Hexb*, *Sall1*, *Tgfbr1, Mef2a, Golm1, Tmsb4x,* and *Tppp*)*,* astrocytes (*NFIA*, *NFIB, GFAP*, *S100B* and *Aqp4*) and oligodendrocytes (*Olig1*, *Olig2*, *Sox10*, *Nkx2.2, Myrf, Zfp488, Cldn11*, *Plp1, Foxo4* , *Cnp*, *Mbp, Mag and Mog*) were also up-regulated in response to maternal infection (Fig. 3, Supplementary Table 1).

### Maternal *H. bakeri* infection enhanced hippocampal LTP in uninfected offspring

In a mouse strain similar to the one we used here, a progressive developmental increase in the capacity to induce hippocampal CA1 LTP has been documented. LTP lasting > 60 min was found in 0% of slices from 2 week-old mice, 26% of slices from 3–4 week-old mice, and 69% of slices from 5 week-old mice^24^. To determine whether maternal *H. bakeri* infection accelerates the development of hippocampal LTP, we used acute hippocampal slices from 21 to 24 day old male pups to record field excitatory postsynaptic potentials (fEPSPs) in the CA1 stratum radiatum, evoked by CA3 Schaffer collateral stimulation.

**Figure 4:**
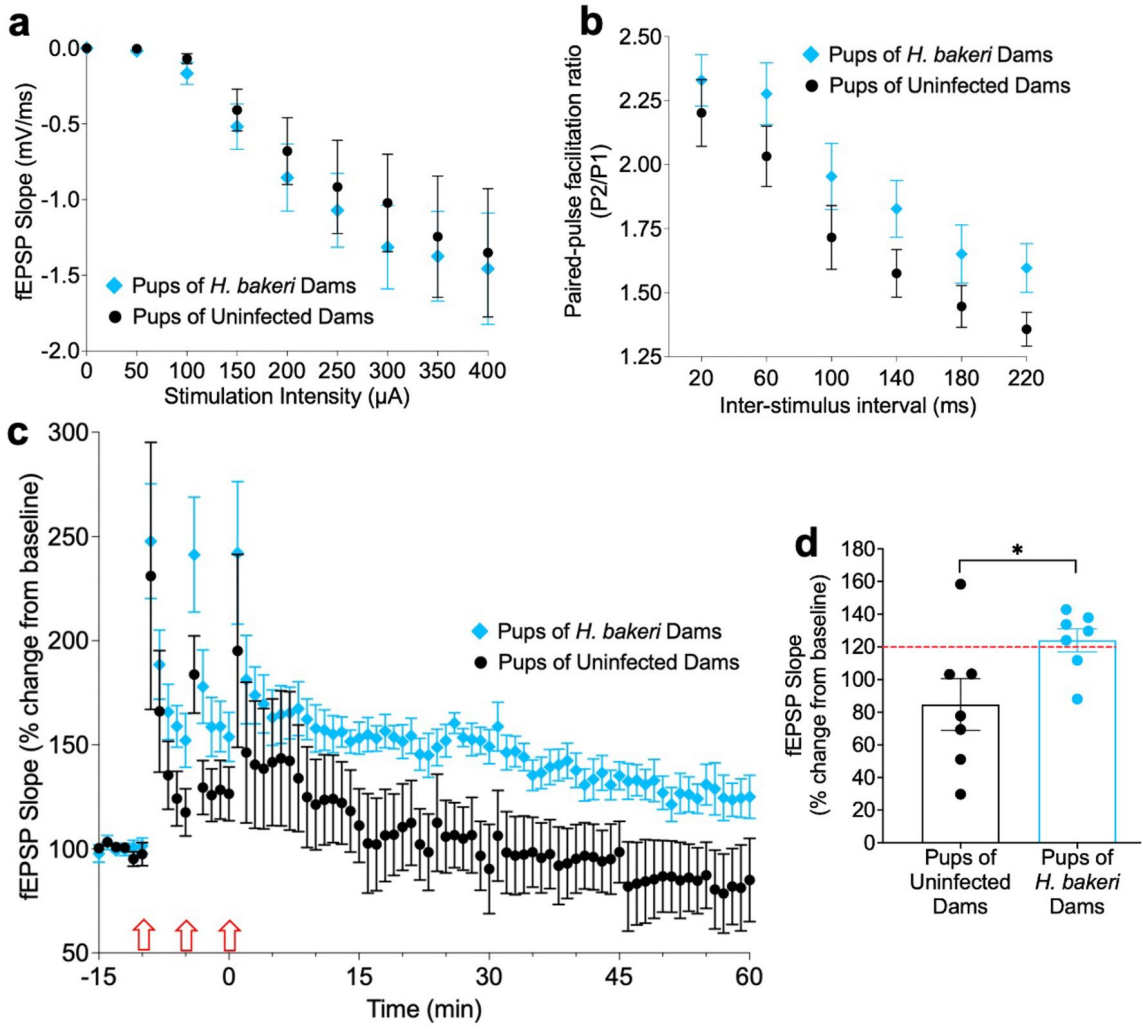
Maternal *H. bakeri* infection enhanced long-term activity-dependent synaptic plasticity at hippocampal CA3–CA1 Schaffer collateral synapses of 3 week-old male offspring. (**a**) Input/output curves showed no differences in basal synaptic transmission between groups. (**b**) Paired-pulse facilitation ratio (PPF) was not altered by maternal *H. bakeri* infection. PPF was obtained by delivering two stimuli with increasing interpulse intervals. PPF was calculated by dividing the second peak slope by the first. (**c**) High-frequency stimulation (HFS)-induced LTP at CA1 was enhanced in juvenile offspring born to *H. bakeri* dams relative to offspring born to uninfected dams (red arrows indicate HFS). (**d**) fEPSP slope measured 60 min after the HFS, indicating maintenance of LTP in 5 of 7 offspring born to *H. bakeri* dams and to only 1 of 7 offspring born to uninfected dams. Values are means $\pm$ SEM, n = 7 offspring per maternal treatment condition (*p < 0.05).

Stimulus input/output (I/O) plots showed no difference in fEPSP slope over increasing stimulation intensity between groups (F_1,12_ = 0.21, p = 0.66; Fig. 4a), indicating that basal synaptic transmission was generally unaltered by maternal infection. Paired-pulse facilitation ratio (PPF), a proxy measure of presynaptic release probability^34^, also showed no significant difference between groups across inter-pulse intervals (F_1,12_ = 2.53, p = 0.14; Fig. 4b), suggesting presynaptic release was also generally unaltered in pups by maternal infection.

LTP was induced by HFS only in pups of infected dams (Fig. 4c,d). In pups of infected dams, the fEPSP slope measured 60 min after the last HFS was 124% ± 7.1% of baseline, a value significantly higher than the fEPSP slope in pups of uninfected dams (84.7% ± 15.9% of baseline) (t = 2.23, df = 12, p = 0.04), indicating an enhanced capacity to induce LTP in response to maternal infection. Also, a higher proportion of slices from pups of infected mothers maintained LTP for > 60 min (5/7 = 71%), compared to pups of uninfected mothers (1/7 = 14%). The data suggest the capacity to induce and maintain LTP is accelerated as a result of maternal *H. bakeri* infection.

### Maternal transfer of *H. bakeri*-specific IgG1 and increased resistance to infection

**Figure 5:**
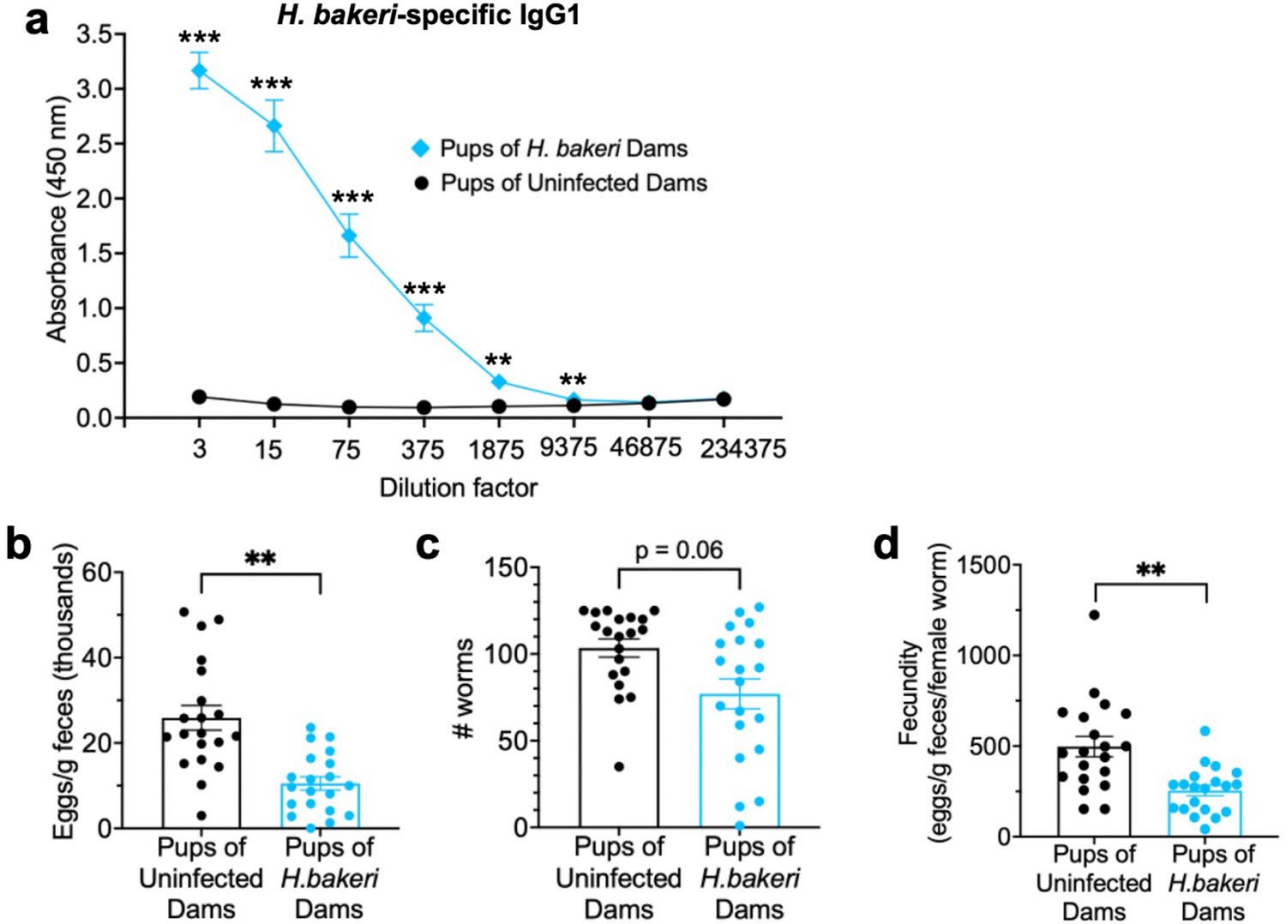
Maternal *H. bakeri* infection resulted in greater resistance to *H. bakeri* infection of their offspring consistent with maternal transfer of parasite-specific IgG1. (**a**) At PD 24, serum was collected from uninfected offspring of *H. bakeri* infected and uninfected dams and *H. bakeri*-specific IgG1 antibody absorbance curves were obtained via ELISA. Uninfected pups of *H. bakeri* infected dams (n = 8) had detectable levels of *H. bakeri*-specific IgG1 antibody while pups of uninfected dams (n = 4) did not. (**b**–**d**) At PD 27, ten male and ten female offspring of *H. bakeri* infected and uninfected dams were infected with 150 *H. bakeri* larvae, and one-month later, (**b**) eggs per gram of faeces, (**c**) worm burden and (**d**) parasite fecundity were obtained as parasitological indicators of infection intensity. *H. bakeri* infection intensity was lower in pups of *H. bakeri* dams compared to pups of uninfected dams. Since no significant sex differences were found between pups, pooled data is shown. Values are means $\pm$ SEM, n = 20 offspring per maternal treatment condition (**p < 0.01, *** *p* < 0.001).

As the maternal immune system influences that of their offspring, and the immune system can strongly influence neurodevelopment and behaviour, the impact of maternal infection on the ability of juvenile male and female offspring to resist a direct *H. bakeri* infection was assessed as an index of peripheral immunity. Serum from PD 24 uninfected offspring of *H. bakeri* infected dams had readily detectable levels of *H. bakeri*-specific IgG1 antibody, whereas pups of uninfected dams did not (Fig. 5a). At PD 27, offspring of *H. bakeri* infected and uninfected dams were experimentally infected with 150 *H. bakeri* larvae. One-month later, infection intensity was significantly lower in pups of *H. bakeri* dams compared to pups of uninfected dams, as indicated by fewer eggs per gram of faeces (p = 0.003; Fig. 5b), worm burden (p = 0.063; Fig. 5c), and fecundity (p = 0.005; Fig. 5d). No effect of pup sex was detected (all p values > 0.17, data not shown).

### Maternal *H. bakeri* infection increased glial cell density in hippocampus of uninfected offspring

**Figure 6:**
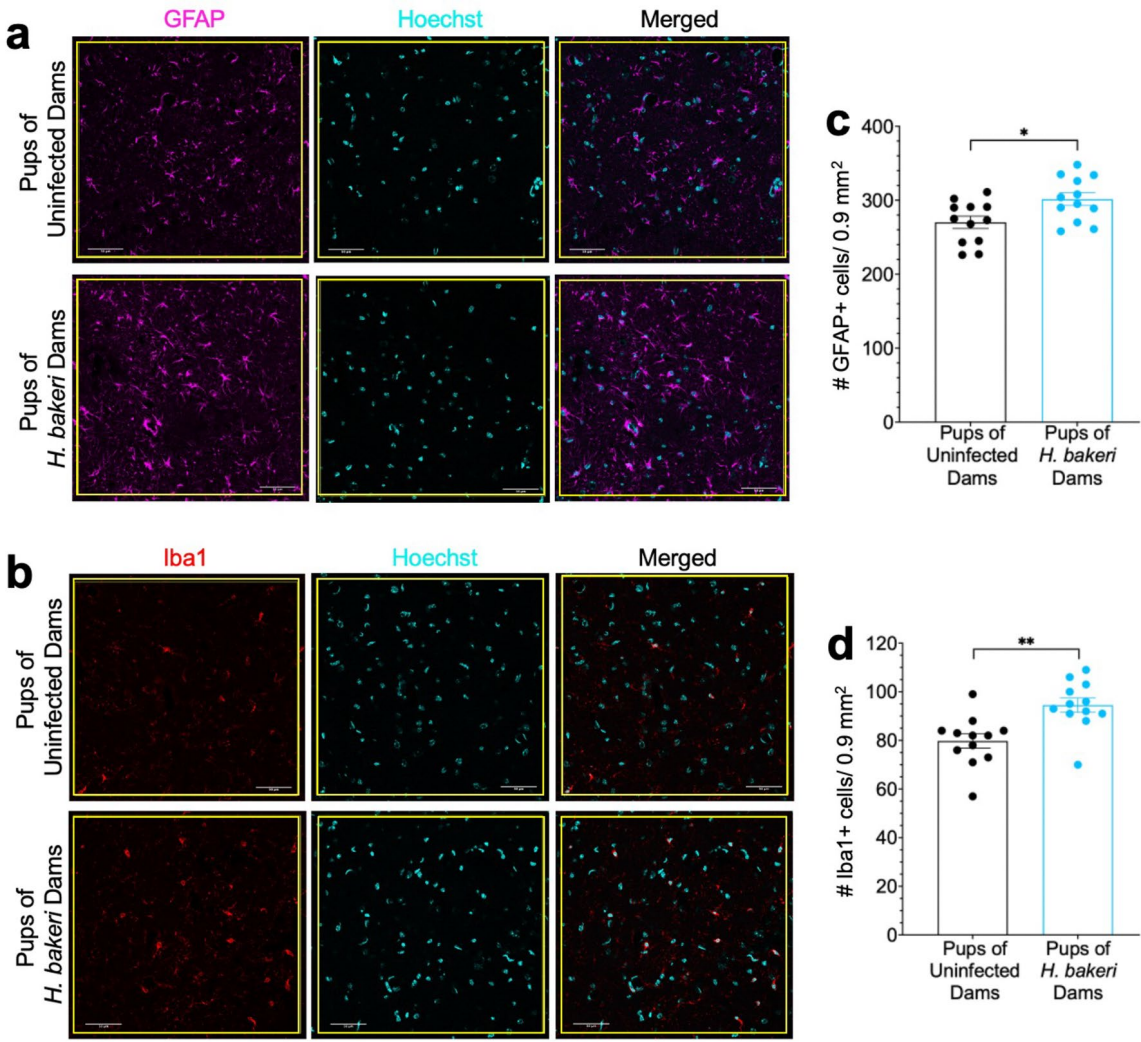
Astrocyte and microglia density are increased in hippocampus of offspring born to *H. bakeri* infected dams. (**a**) Astrocytes were detected with an antibody directed against GFAP (magenta) and (**b**) microglia were detected with an antibody directed against Iba1 (red). Cells within 0.1 mm^2^ yellow boxes (shown below) were counted. Cell nuclei were stained with Hoechst dye (cyan). Scale bar: 50 μm. (**c**) GFAP positive cells and (**d**) Iba1 positive cells were counted in three × 0.1 mm^2^ boxes per hippocampal section, and a total of three hippocampal sections per mouse were assessed (# cells/0.9 mm^2^/mouse). Since no significant sex differences were evident, pooled female and male data are shown. Values are mean ± SEM, *n* = 12 offspring per maternal treatment condition (*p < 0.05, **p < 0.01).

**Figure 7:**
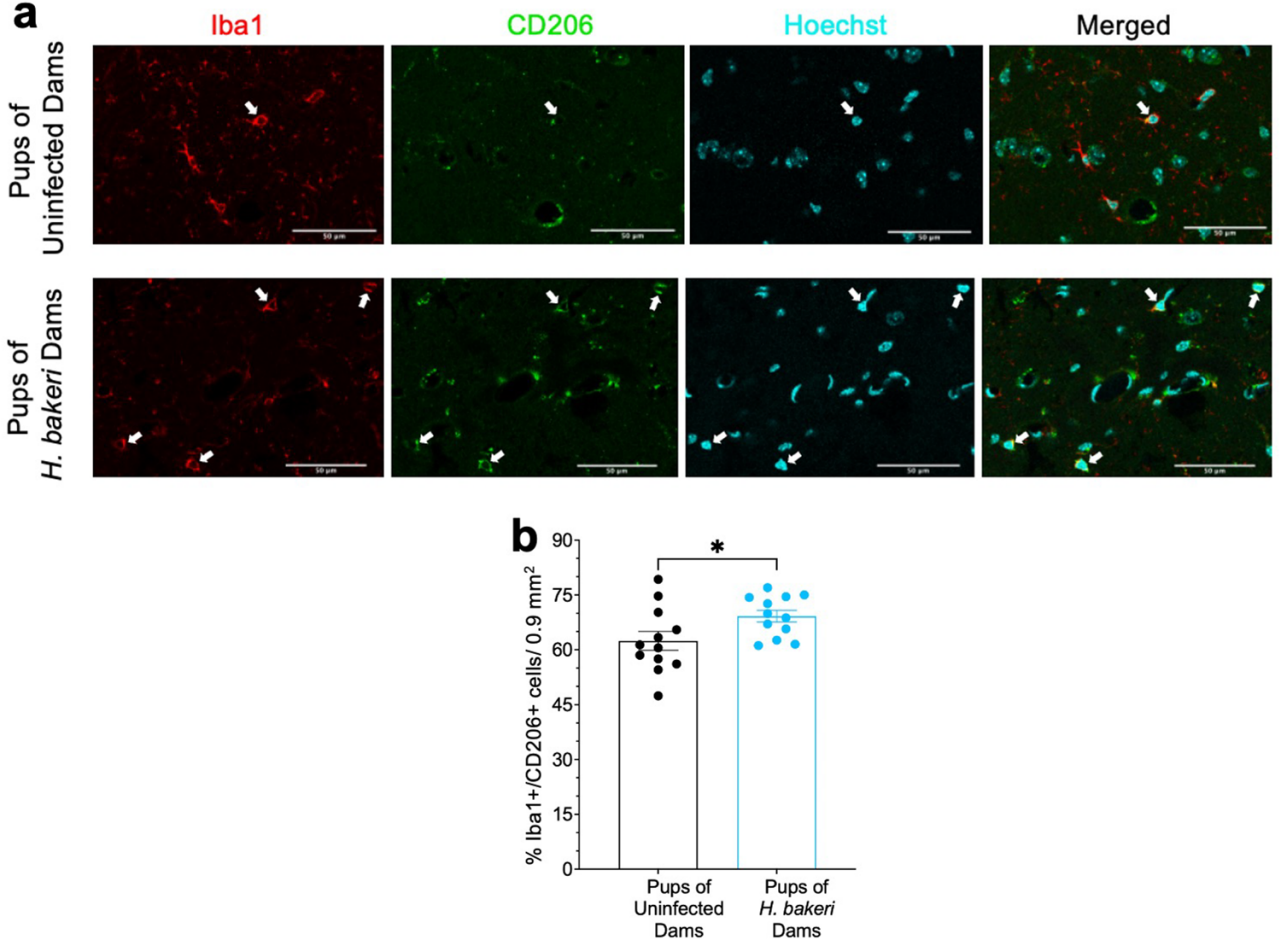
Percent of microglia positive for CD206 is increased in hippocampus of offspring born to *H. bakeri* infected dams. (**a**) Double immunofluorescence labeling of Iba1 + /CD206 + microglia cells in hippocampus of offspring born to *H. bakeri* infected or uninfected dams. Microglia were detected by Iba1 antibody (red). To detect CD206 positive microglia, the Mouse Macrophage Mannose Receptor/CD206 antibody was used (green), and double labelled Iba1 + /CD206 + cells were assessed (indicated by white arrows). Cell nuclei were stained with Hoechst dye (cyan). Scale bar: 50 μm. (**b**) All Iba1 + and Iba1 + /CD206 + cells were counted in three × 0.1 mm^2^ boxes per hippocampal section, and a total of three hippocampal sections per mouse were assessed (# cells/0.9 mm^2^/mouse). The percent of Iba1 + /CD206 + cells relative to the total number of Iba1 + cells was calculated. Since no significant sex differences were evident, pooled female and male data are shown. Values are mean ± SEM, *n* = 12 offspring per maternal treatment condition (*p < 0.05).

To determine if maternal *H. bakeri* infection altered the neuroimmune system in the hippocampus of PD 22 uninfected offspring, brain sections were immunohistochemically labelled for astrocytes and microglia with antibodies against GFAP and Iba1 respectively. To gain insight into the functional role of microglia, we also immunostained for CD206, a microglial cell surface protein typically increased in response to Th2 cytokines^30,31,35^. Cell density was assessed in the dorsal hippocampus, with a focus on the CA3-CA1 region (Supplementary Fig. 3). Pups of *H. bakeri* infected dams had higher density of cells positive for GFAP (F_1,20_ = 6.22, p = 0.02; Fig. 6a,c) and Iba1 (F_1,20_ = 11.81, p = 0.003; Fig. 6b,d), and a higher percentage of Iba1 + /CD206 + cells (F_1,20_ = 5.09, p = 0.04; Fig. 7a,b), relative to pups of uninfected dams. Pup sex did not affect astrocyte or microglia density, nor the percentage of Iba1/CD206 double positive cells (p > 0.48, data not shown). Together with gene expression data, the results suggest maternal *H. bakeri* infection promotes a Th2/Treg biased immune response in the hippocampus of uninfected offspring.

## Discussion

Here we used a mouse model to examine the influence of a maternal GI nematode infection on hippocampal gene expression, LTP and the neuroimmune system, as well as resistance to direct infection of 3 week-old juvenile offspring. We observed four key consequences of maternal infection: (1) earlier onset of the capacity to induce hippocampal LTP, with changes in gene expression suggesting this may be due to accelerated maturation of glutamatergic synapses; (2) higher levels of hippocampal gene expression in neurogenesis and gliogenesis pathways, and higher levels of gene expression associated with oligodendrocytes and myelination; (3) greater resistance to *H. bakeri* infection, evidenced by lower worm burden and parasite fecundity, consistent with maternal transfer of parasite-specific IgG1 to the serum of uninfected offspring; and (4) an increase in the density of hippocampal microglia and astrocytes, a higher percentage of CD206 positive microglia, and increased expression of the TGF-β signaling pathway involved in immune regulation. Together, the data suggest immune stimuli from the *H. bakeri* infected mother are transferred to the uninfected offspring, extend to their brain, and result in a Th2/Treg-biased neuroimmune response that underlies accelerated hippocampal maturation as evidenced by enhanced LTP, increased expression of genes associated with neurogenesis, gliogenesis, and myelination, and enhanced spatial memory^12^.

Maternal *H. bakeri* infection enhanced or accelerated the developmental capacity for LTP induction in uninfected offspring, which may be due to the observed changes in gene expression. As the capacity for hippocampal LTP is believed to strongly correlate with successful spatial memory formation^19^, this is consistent with the enhanced spatial memory we previously detected in offspring of *H. bakeri* infected dams^12^. To our knowledge, this has not been described in other maternal infection models, although similarly to our observations here, exposure to exercise during early life enhances LTP and spatial memory^36^. In contrast, maternal exposure to *Escherichia coli* lipopolysaccharides^5,37^ or stress^38^, both of which are known to induce neuroinflammation in offspring^3,39^, impairs hippocampal LTP and spatial memory in offspring.

Consistent with altered LTP, we found increased expression of genes associated with glutamatergic synapses and the MAPK signalling pathways associated with LTP, in addition to those associated with AMPAR and NMDAR subunits, CAMKII and PKC, each of which may contribute to LTP induction and maintenance. GluR1 AMPAR subunits are critical for LTP and memory retention^40^. Exposure of pregnant mice to a bacterial mimic reduced GluR1 in offspring hippocampi and impaired spatial memory^41^, whereas increased hippocampal GluR1 improved spatial memory in rats^42,43^. Hippocampal LTP also relies on NR2A-containing NMDARs^44^. Exposure of pregnant mice to a viral mimic decreased NR2A in offspring hippocampi and impaired spatial memory^45^. In contrast to inflammatory maternal infections, maternal *H. bakeri* infection resulted in increased expression of the GluR1 and NR2A genes, *Gria1 and Grin2a,* whereas expression of the GluR4 gene, *Gria4,* was reduced. As *Gria1* and *Grin2a* subunit expression increases during normal neurodevelopment, and *Gria4* decreases^46–48^, our results suggest that maternal *H. bakeri* infection accelerates hippocampal glutamate synapse maturation.

CaMKII and PKC are calcium-dependent-kinases with important roles in LTP; both are linked to positive regulation of GluR1 AMPAR subunit phosphorylation, which increases AMPAR conductance and postsynaptic responsiveness^20–22^. Injection or overexpression of CAMKII^49–51^ or PKC^52,53^ enhances LTP and spatial memory. CaMKII has four distinct isoforms (α, β, γ, and δ)^54^, and PKC consists of at least 10 isoforms, including the classical subfamily of PKC isozymes (PKCα, PKCβ and PKCγ)^55^. In response to maternal *H. bakeri* infection, we detected increased expression of CaMKII α and β genes (*Camk2a* and *Camk2b,* respectively)*,* and the PKCα gene, *Prkca*. Loss of CaMKIIα, CaMKIIβ^56–59^ or PKCα^55^ results in severe impairment of LTP and spatial memory. Taken together, higher expression of *Gria1* and *Grin2a,* lower expression of *Gria4,* with higher expression of *Camk2a*, *Camk2b* and *Prkca* would be expected to promote LTP, as demonstrated here, and facilitate spatial memory in response to maternal infection, as previously reported^12^.

The increased gene expression associated with neurogenesis pathways may also contribute to enhanced spatial memory in juvenile offspring^12^. In the adult brain, neurogenesis in the hippocampal dentate gyrus contributes to spatial memory^60^, and maternal physical exercise increases offspring hippocampal neurogenesis and spatial memory, via brain derived neurotrophic factor (BDNF)^61–64^. BDNF is a key positive modulator of LTP and neurogenesis^65^. Here we found increased hippocampal *Bdnf* expression in response to maternal infection (p = 0.015, adjusted p value = 0.067). Further studies are required to determine how enhanced hippocampal neurogenesis may contribute to the enhanced spatial memory detected in juvenile offspring of *H. bakeri* infected dams^12^.

Our findings support the hypothesis that maternal GI nematode infection alters hippocampal function and spatial memory in developing uninfected offspring by transfer of maternal immunity, as offspring immunity strongly influences neurodevelopment and behaviour^8^. Maternal immune antibodies and cells are transferred to young offspring via nursing, offering protection from pathogens and shaping immune system maturation^10^. For example, via nursing, *H. bakeri*-specific IgG1 antibody is transferred from infected mothers to pre-weaned 10-day old neonates, providing protection against *H. bakeri*^26^, and maternally derived Th2 CD4 + T cells are transferred from *Nippostrongylus brasiliensis* infected mothers to offspring, providing long-lasting cellular immunity against direct infection with this nematode^9^. Our hypothesis that the Th2/Treg immune response in the *H. bakeri* infected dam is mimicked in 3-week-old uninfected weaned offspring is supported by *H. bakeri*-specific IgG1 in the serum of the uninfected offspring of infected dams. Further, fewer adult worms with reduced fecundity in *H. bakeri* infected offspring of infected dams, compared with infected offspring of uninfected dams, indicates heightened resistance and that the functional immunity induced by maternal infection parallels that seen during a secondary challenge in this mouse model^11^.

Additionally, *H. bakeri*-specific antibodies in offspring serum parallel a Th2/Treg biased neuroimmune response in the hippocampus, which may alter glial differentiation, development and function. This may support LTP, neurogenesis, gliogenesis, and myelination, and contribute to improved spatial memory^12^. We previously reported that whole brain samples of uninfected seven-day old male neonates of *H. bakeri* infected dams had up-regulated expression of IL4 and TGF-β genes^15,27^. These cytokines are hallmarks of *H. bakeri* infection^11^, and vital for the regulation of brain immunity with downstream effects on LTP, neurogenesis, and spatial memory^66–71^. Consistent with this, we detected higher expression of the TGF-β signaling pathway and of *Tgfb2* and *Tgfbr1* genes but downregulation of the *Tgfb3* gene in the hippocampus of uninfected juvenile offspring. Of the three isoforms of TGF-β, encoded by the genes *Tgfb1-3*, TGF-β2 regulates hippocampal synaptic plasticity^68^ and neurogenesis^69^, and TGF-β2 knockout mice exhibit synaptic and cognitive dysfunction^72,73^. Further, gene expression and protein levels of TGF-β2 and its receptor, TGF-β receptor 1 (TGF-βR1), are higher in IL-4 induced microglia^74^, which are associated with immune regulation and memory^75^. In contrast, TGF-β3 promotes Th17 cell differentiation and the pathogenesis of autoimmune diseases^76,77^. The up-regulated expression of both *Tgfb2* and *Tgfbr1* genes*,* and downregulation of the *Tgfb3* gene suggests a TGF-β immunoregulatory response in the hippocampus of the uninfected offspring.

TGF-β is also critical for differentiation, development and function of neurons and glia^69,78^. The higher density of microglia and higher expression levels of microglia-specific genes (*Hexb*, *Sall1*, *Tgfbr1, Mef2a, Golm1, Tmsb4x,* and *Tppp*), higher density of astrocytes and higher expression levels of astrocyte-specific genes (*Nfia*, *Nfib, Gfap*, *S100B* and *Aqp4*), and greater percentage of CD206 positive microglia in the hippocampus of offspring of *H. bakeri-*infected dams indicate a hippocampus responding to Th2/Treg factors. CD206, like TGF-β2 and TGF-βR1, is typically increased by microglia responding to IL-4, suggesting a microglia phenotype associated with immune regulation^30,31,35,74^. Expression of *Tgfbr1, Hexb, Golm1*, and *Sall1* increase with microglia maturity, and maturation is dependent on TGF-β signaling^78,79^ which was up-regulated by maternal infection. *Nfia* and *Nfib* contribute to astrocyte development, *Gfap*, *S100B* and *Aqp4* are markers of mature astrocytes^80^, and *Aqp4* is important in TGF-β-associated immunoregulation^81^.

TGF-β signaling is also critical for oligodendrogenesis and developmental myelination^70^. In response to maternal *H. bakeri* infection, we detected higher expression of 13 key genes associated with oligodendrocytes and myelination. *Olig1*, *Olig2*, *Sox10*, *Nkx2.2, Myrf, Zfp488* and *Cldn11*, are necessary for oligodendrocyte differentiation and myelination during development^32,82^. *Plp1*,* Foxo4* and *Cnp* are expressed during oligodendrocyte differentiation, and mature myelinating oligodendrocytes express *Mbp*,* Mag* and* Mog*^32^. Delayed hippocampal myelination leads to impaired excitatory synaptic transmission and cognitive dysfunction^4,83,84^, whereas early developmental hippocampal myelination promotes excitatory synaptic transmission and cognitive function, including spatial memory^85^. Thus, accelerated oligodendroglial maturation and myelination in the developing hippocampus via TGF-β signaling could further contribute to enhanced LTP and spatial memory^12^ observed in juvenile offspring in response to maternal *H. bakeri* infection.

It is worth highlighting the contrasting consequences of maternal bacterial and viral infections with intestinal nematode infections. Maternal bacterial or viral infections induce a strong Th1/Th17 immune response in the mother that extends to the offspring brain, and is associated with microglia- and astrocyte-mediated neuroinflammation. If prolonged, neuroinflammation drives oligodendroglial apoptosis, delays myelination, and impairs LTP and neurogenesis, ultimately resulting in the emergence of ASD-like behaviours and cognitive impairments in the offspring^3–5,83,84,86^. Conversely, our data demonstrate that maternal intestinal nematode infection induced a Th2/Treg immunoregulatory environment in the developing hippocampus, that was associated with upregulated genetic markers of neurogenesis, gliogenesis and myelination, and enhanced LTP and spatial memory^12^. We hypothesize that these differences result from the transfer of Th2/Treg-specific immune molecules from the nematode-infected mother but do not exclude other pathogen-related differences in the maternal microbiome that may affect microbial colonization of the offspring^87,88^. Regardless, if the observed changes induced by maternal nematode infection persist as pups grow, and if damaging neurological changes do not occur, this raises the possibility that GI nematodes might be important for proper brain development and function, and may provide a promising avenue for preventing inflammation-associated neurodevelopmental disorders.

We acknowledge the following limitations. Our electrophysiology experiments recorded LTP in male but not female offspring. We hypothesize that similar results would have been seen in females as sex did not affect spatial memory of juvenile offspring of *H. bakeri* infected mothers^12^ or hippocampal gene expression or microglia/astrocyte density. Also, data from only the first slice tested per animal was used in our LTP experiment as subsequent slices were of lower quality, limiting our sample size, however, this avoided pseudoreplication. We also acknowledge that new hypotheses are based on gene expression data. Thus, it will be important to confirm our gene expression findings by assessing protein levels. For instance, it will be of great interest to perform unbiased stereology on BrdU/NeuN-double-labeled cells in the dentate gyrus to determine if maternal *H. bakeri* infection enhances hippocampal neurogenesis, as well as to assess cell density of oligodendrocytes and myelination, and levels of key cytokines (IL-4 and TGF-β) in this model. Finally, caution is needed in interpreting the function of microglia and astrocytes given their high plasticity.

To the best of our knowledge, this is the first study to show that a maternal GI nematode infection promotes hippocampal LTP and upregulates genetic markers associated with neurogenesis, gliogenesis and myelination in the uninfected juvenile offspring, possibly through transfer of a Th2/Treg immune phenotype from the infected dam that protects the offspring from direct infection and extends to their developing brain. These findings identify possible mechanisms underlying our previous observation of enhanced spatial memory in two and three week-old offspring exposed to *H. bakeri* maternal infection^12^. These positive effects on neurodevelopment and cognition identify a potential unappreciated benefit of maternal GI nematode infection. Given the immunoregulatory nature of this parasite, that extends to the offspring, our findings may be valuable in efforts to prevent the development of inflammation-associated neurodevelopmental disorders, like ASD.

## Methodology

### Experimental design

We compared juvenile offspring of *H. bakeri* infected versus uninfected dams.

### Mice and parasites

38 primiparous 8 week-old timed pregnant (GD 4) outbred CD-1 mice were received at McGill Macdonald Campus’ Animal Facility from Charles River Laboratories, Quebec, Canada. Each dam with her litter was housed individually in a Nalgene cage (Fisher Scientific, Canada) at 21–23 °C, 40–60% relative humidity and a 12 h light and dark cycle. Mice had ad libitum access to a 2920X Teklad rodent diet (18% crude protein, 5% crude fat, 5% crude fiber). Within each of the eight staggered groups of dams received, dams were randomized into uninfected and *H. bakeri* infected groups, with a total of 18–20 dams per group. Using standard *H. bakeri* protocols^89^, infective third-stage larvae (L3) were obtained by fecal culture of stock parasites maintained in outbred CD-1 mice. Dams in the *H. bakeri* group were infected using an oral gavage needle with 100 ± 3 L3 suspended in 0.1 mL distilled water on GD 7, 12, 17, and PD 3, 8 and 13. Uninfected dams received 0.1 mL distilled water via oral gavage at the same frequency. Given that *H. bakeri* eggs released into the environment develop into L3 after 7 days, all cages were cleaned every 5 days to ensure offspring could not ingest L3. Dams were weighed on GD 7, 12 and 17 to ensure infection did not result in weight loss. Following weaning (PD 20), dams were euthanized and necropsied and successful infection of dams was confirmed by noting presence of adult worms in the small intestine.

Pups were born on GD 19 and litter size was recorded. At PD 20, pups were weaned, sexed, given a unique identifier, and body mass and length from the top of the head to the base of the tail recorded. On PD 21, a subset of pups were transported to the Montreal Neurological Institute’s Animal Facility for the experiments outlined below. Pups within each litter were randomly selected for each experiment. At euthanasia, experimental pups were necropsied and intestines were examined for adult *H. bakeri*. This confirmed that the offspring had not been accidentally infected. Pups not used for this study were assigned to a separate study.

### Compliance with guidelines for research with experimental animals

This study (protocol #2000-4601) was approved by the McGill University Animal Care Committee according to the guidelines of the Canadian Council on Animal Care. All methods were carried out in accordance with relevant guidelines and regulations, and the study was carried out in compliance with ARRIVE guidelines (https://arriveguidelines.org).

### Gene expression study

#### Tissue samples

On PD 23, three male and six female offspring from uninfected dams and five male and five female offspring from *H. bakeri* dams (no more than one pup/sex/dam) were decapitated without anesthesia, as anesthesia can influence gene expression in the brain. The brain was rapidly removed and using iced cold artificial cerebrospinal fluid (aCSF), hippocampi were rapidly removed bilaterally, immediately flash frozen in liquid nitrogen, then stored at − 80 °C. Hippocampal samples were sent to Genome Quebec for total RNA extraction and sequencing and FASTQ files were obtained. No pooled samples were used.

#### Homogenization

900 µL of RNeasy Plus Universal Mini Kit provided lysis buffer reagent (i.e. QIAzol) was added to previously weighted tissue (10–15 mg). Homogenization was done using a QIAGEN TissueLyser II with 5 mm stainless beads, for 2 cycles of 30 Hz × 2 min plus 1 cycle of 30 Hz × 1 min.

#### Extraction

Total RNA extraction was performed using the RNeasy Plus Universal mini kit (QIAGEN, cat.73404) according to the manufacturer's instructions. RNA was eluted in 35 µL buffer provided with the extraction kit. RNA quality was determined by the RNA Integrity Number (RIN), measured by 2100 Bioanalyzer (Agilent Technologies) using RNA 6000 Nano kit, following the manufacturer’s protocol.

#### Library preparation

Libraries were generated from 250 ng of total RNA using the Illumina^®^ Stranded mRNA Prep, Ligation Kit (Illumina), as per the manufacturer’s recommendations. Libraries were quantified using the KAPA Library Quanitification Kits—Complete kit (Universal) (Kapa Biosystems). Average size fragment was determined using a LabChip GXII (PerkinElmer) instrument.

#### Sequencing

Libraries were normalized and pooled and then denatured in 0.02 N NaOH and neutralized using HT1 buffer. The pool was loaded at 175 pM on an Illumina NovaSeq 6000 S4 lane using Xp protocol as per the manufacturer’s recommendations. The run was performed for 2 × 100 cycles (paired-end mode). A phiX library was used as a control and mixed with libraries at 1% level. Base calling was performed with RTA v3. Program bcl2fastq2 v2.20 was then used to demultiplex samples and generate FASTQ reads.

#### Gene expression analysis

Raw reads were aligned to the mouse GRCm38 reference transcriptome using the Kallisto software^90^ (version 0.46.1, minimum quality score = 25). Transcripts were filtered to remove those with low abundance and low variability across all samples (abundance < 4 counts, removed 15th percentile with lowest variability). Counts were normalized and converted into log2-counts-per-million (logCPM) using the Relative log expression normalization method as implemented in the edgeR R package^91^ (version 3.38.4). Differential expression analysis (DEA) of the logCPM values was conducted with the edgeR R package to identify genes with significantly different expression between offspring of *H. bakeri-*infected or uninfected mothers (adjusted p-value < 0.05, FDR method). Since principal component analysis showed no pattern with respect to sex in the top components and DEA of male versus female yielded only 10 differentially expressed genes (DEGs), offspring sex was excluded from the analysis. Hypergeometric tests were used to identify gene sets (KEGG and GO BP) that were significantly overrepresented in the list of DEGs (adjusted p-value < 0.05, FDR method). Analyses were performed twice, once using the list of DEGs with positive log2FC, and once using the list with negative log2FC, to identify overrepresented pathways in the list of up-regulated and down-regulated DEGs. All gene expression analysis was conducted using the ExpressAnalyst software (https://www.expressanalyst.ca/), a web-based platform for processing, analyzing, and interpreting RNA-sequencing data^92^.

### Long-term potentiation study

#### Brain slice preparation

On PD 21–24, seven male offspring from *H. bakeri* infected or uninfected dams (no more than one pup/dam) were decapitated without anesthesia and the brain was immediately removed and submerged in ice-cold oxygenated (95% O_2_/5% CO_2_) artificial cerebrospinal fluid (aCSF) (in mM: 120 NaCl, 3 KCl, 2 MgSO_4_, 2 CaCl_2_, 1.2 NaH_2_PO_4_, 23 NaHCO_3_, 11 glucose) for one min. The brain was placed on an iced-cold platform with aCSF and both hippocampi were rapidly removed. Transverse hippocampal slices (400 μm) were cut with a tissue chopper (MclLwain, TC752). Approximately three slices from the middle third of the hippocampus were obtained from each hemisphere. Slices were kept in chilled and oxygenated aCSF and the hippocampal CA3 region was removed with a scalpel. Slices were then placed in a humidified and oxygenated (95% O_2_/5% CO_2_) interface chamber (Digitimer, BSC2-2) perfused (0.15 mL/min) with aCSF at 28–30 °C. Slices were left to recover for at least 1.5 h before recording.

#### Electrophysiology

Extracellular recording pipets (1.5–3 MΩ) encasing a chlorinated silver wire stripped at the tip were pulled from borosilicate glass capillary tubing (Warner Instrument, Hamden, CT), filled with 4 M NaCl and placed in stratum radiatum of CA1 to record field excitatory postsynaptic potentials (fEPSPs). Synaptic events were evoked by Schaffer collateral stimulation by placing a concentric bipolar stimulating electrode (FHC Inc., Bowdoin, ME) (~ 500 µm lateral from the recording electrode) in stratum radiatum of area CA1.

Slices were stimulated every 10 s and an input–output (I/O) curve generated by measuring the slope (mV/ms) of the extracellular field excitatory postsynaptic potentials (fEPSPs) in response to increasing stimulus intensities (ranging from approx. 0–400 μA). Stimulus intensity was increased to the point where a population spike was just detectable in the fEPSP record, and the test response was then set at 50% of this stimulus intensity. Paired-pulse facilitation ratio (PPF) was then assessed as an increase in the size of the synaptic response to a second pulse delivered within a short interval of time following the first pulse. PPF is a form of short-term synaptic plasticity that results primarily from presynaptic mechanisms and is generally explained as an increase in the probability of vesicular release during the second stimulus, arising from prior accumulation of residual calcium^34^. PPF can be used to help determine if any differences observed in LTP are associated with presynaptic involvement^34^. To this aim, paired stimuli to the Schaffer collaterals were applied at increasing interpulse intervals (ranging from 20 to 220 ms at 40 ms increments) and the paired-pulse facilitation ratio was determined as the slope of the second fEPSP divided by that of the first fEPSP. Following PPF, baseline responses to stimulation at a frequency of 1 pulse every 20 s were recorded. Once a stable baseline response had been established for 30 min, a high frequency stimulation (HFS) (3 × 100 Hz for 1 s, with 10 s between each 100 Hz train; repeated 3 × at 5 min intervals) was applied, and responses were measured every 20 s for 60 min after the HFS. Data were analyzed using Clampfit software (Version 10.7). Fiber volley amplitude (which is an indication of the presynaptic action potential arriving at the recording site) was measured during the I/O curve and throughout the LTP experiment to ensure it remained stable. The initial slope of the fEPSP was used as a measure of synaptic strength as this is preferred over the potential amplitude to avoid contamination of the fEPSP by a population spike. The baseline response for the LTP experiments was calculated as the average response generated 5 min before HFS. All values were then converted to a percent change from the average baseline. A slice was considered potentiated if it remained ≥ 120% of baseline at 60 min^24^. Of note, there was only access to one interface recording chamber, preventing synchronous testing of multiple slices per mouse. Data from only the first slice tested per animal was used as subsequent slices were of lower quality, avoiding pseudoreplication.

#### Statistics

Data was analyzed in GraphPad Prism (Version 10.0.2) to compare offspring from *H. bakeri* infected dams with offspring from uninfected dams. Mixed models were used to test for differences in the I/O curve, PPF and LTP data between groups, followed by Sidak multiple comparisons test. Unpaired t-tests were conducted to compare the response between groups generated at 60 min following HFS. Values are presented as means ± SEM. The significance level was set at 0.05.

### Resistance study

#### Serum collection and ELISA for *H. bakeri* specific IgG1

On PD 24, eight offspring (4 per sex) from *H. bakeri* infected and four offspring (two per sex) from uninfected dams (no more than one pup/sex/dam) were anesthetized with isoflurane and blood samples were collected via cardiac puncture. Serum was stored at − 20 °C.

*Heligmosomoides bakeri*-specific IgG1 antibody absorbance curves were obtained via enzyme-linked immunosorbent assays (ELISA). *H. bakeri* excretory-secretory antigen (HES) was made using the well-established protocol by Stevenson et al.^93^. An ELISA plate (Nunc Maxisorp) was coated with 50 μL HES diluted in PBS to 1 μg/mL overnight at 4 °C. The plate was washed 5 times with ELISA wash buffer (PBS + 0.05% Tween-20) and then blocked with 2X Ebioscience Assay Buffer A (PBS with 1% Tween-20 and 10% BSA) for 2 h at room temperature (RT). The plate was then washed 5 times with ELISA wash buffer. 100 μL serum was added to each well using fivefold serial dilutions, with a total of 8 serial dilutions (the starting dilution was threefold). Serial dilutions were done using 1× Ebio Assay Buffer A. Serum was incubated for 2 h at RT. The plate was washed 5 times with ELISA wash buffer. Primary Antibody (Rat Anti-Mouse IgG1-BIOTIN Clone SB77E: Southern Biotech #1144-08) was diluted 5000 × in 1 × Ebio Assay Buffer and 100 μL was added to each well and incubated for 1 h at RT. The plate was washed 5 times with ELISA wash buffer. 100 μL of Secondary Antibody (Streptavidin-HRP: Southern Biotech #7100-05) was diluted 1000 × in 1 × Ebio Assay Buffer and 100 μL was added to each well and incubated for 1 h at RT. Plate was washed 10 times with ELISA wash buffer. 100 μL of TMB substrate solution was added and the reaction was stopped by adding 100 μL 1 M sulfuric acid after 10 min. Plate readout was at 450 nm. The reference wavelength of 570 nm was used (values read at 570 nm were subtracted from those read at 450 nm, giving the absorbance value).

#### *H. bakeri* infection intensity of offspring

On PD 27, ten offspring per sex from five *H. bakeri* infected and five uninfected dams were infected using an oral gavage needle with 150 ± 3 L3 *H. bakeri* suspended in 0.1 mL distilled water. At 36 days post infection, for a 20 h period, each mouse was placed into an individual wire-bottomed cage which allowed for collection of fecal pellets. Drinking water was provided ad libitum but food was withheld. Fecal pellets were collected and the McMaster technique was used to determine egg production expressed as eggs per gram of faeces (EPG). At 38 days post infection, mice were euthanized using isoflurane, followed by CO_2_, and intestines were collected. The number of male and female worms were counted (i.e. worm burden) and the fecundity of female worms of *H. bakeri* were determined as parasitological indicators of infection intensity.

#### Statistics

Statistical analyses were performed in R statistical software 4.2.3, and figures were produced using GraphPad Prism V9. To assess *H. bakeri*-specific IgG1 antibody absorbance, where we had repeated measures across different dilution factors, models were built with maternal treatment condition (*H. bakeri* infected vs. uninfected), offspring sex (male vs. female) and dilution factor as fixed factors, and the identity of the mouse as a random factor. EPG, fecundity and worm burden, models were built with maternal treatment condition (*H. bakeri* infected versus uninfected) and offspring sex (male versus female) as fixed factors, and dam as a random factor to account for pseudoreplication. Non-significant interactions between fixed effects were excluded from models.

Linear mixed models were built to assess *H. bakeri*-specific IgG1 antibody absorbance, EPG and fecundity using the lme function in the nlme package^94^. As worm burden was a discreate and overdispersed variable, a negative binomial generalized linear model was built using the glmer.nb function in the lme4 package^95^. Where necessary, post hoc pairwise comparisons were performed using the emmeans function (emmeans package^96^) with a Tukey correction. Normality, independence and homogeneity of variances of mixed models were assessed using fitted residuals from the plotresid function (RVAideMemoire package^97^), and the DHARMa package^98^. Values are presented as means ± SEM. The significance level was set at 0.05.

### Neuroimmune study

#### Tissue preparation

On PD 22, six offspring per sex from *H. bakeri* infected or uninfected dams (no more than one pup/sex/dam) were anesthetized with intraperitoneal injection of avertin (600 mg kg^–1^ body weight) and then transcardially perfused with ice cold 1 × PBS followed by 4% paraformaldehyde in PBS w/v. Collected brains were fixed with 4% PFA overnight and cryoprotected in 30% sucrose for 24 h. Brains were then embedded in OCT medium and stored at − 80 °C. Using a cryostat (Leica CM3050 S), serial coronal sections (20 μm thick) of the brain were obtained, with a focus on the dorsal hippocampus, and mounted on microscope slides.

#### Immunohistochemistry

Hydrophobic pen (ImmEdge Pen, Vector Laboratories) was used to create a water-repellent barrier to keep reagents localized on tissue sections. Tissue sections were hydrated in 1 × PBS and then incubated in blocking solution (0.03% Triton X-100, 3% heat-induced horse serum (HIHS) and 3% BSA in 1 × PBS) for 1 h at RT. Primary antibody was diluted in new blocking solution, added to the tissue sections and incubated at 4 °C overnight. Tissue sections were washed with 1 × PBS three times for 10 min each. Secondary antibody was diluted in 3% HIHS, 3% BSA in 1 × PBS and added to the tissue sections and incubated for 1 h at RT. These steps were then repeated for the second and third primary and secondary antibodies. Once the final secondary antibody had been applied, tissue sections were washed with 1 × PBS for 10 min followed by staining with Hoechst (1:10,000) in 1 × PBS for 10 min. Two additional 10 min washes in 1 × PBS were performed before tissue sections were air dried and mounted (Dako Fluorescence Mounting Medium, S3023; Agilent). Negative controls that omitted the primary antibodies were included.

#### Antibodies

The following antibodies were used for immunofluorescent staining: Glial Fibrillary Acidic Protein (GFAP) antibody (AB5541, 1:500; Millipore) to detect astrocytes; Ionized Calcium Binding Adapter Molecule 1 (Iba1) antibody (019-19741, 1:500; Wako) to detect microglia. We also stained with Mouse Macrophage Mannose Receptor/CD206 antibody (AF2535, 1:40; R&D Systems), a cell surface protein that is typically increased in response to Th2 cytokines^30,31,35^. This allowed us to detect double stained Iba1 + /CD206 + cells. For secondary antibodies, we used Alexa Fluor 647 Goat Anti-Chicken IgY (A21449, 1:500; ThermoFisher Scientific), Alexa Fluor 555 Donkey Anti-Rabbit IgG (A31572, 1:500; ThermoFisher Scientific) and Alexa Fluor 488 Donkey Anti-Goat IgG (A11055, 1:500; ThermoFisher Scientific).

#### Image capture and analysis

Confocal microscope (Leica SP8) was used to image three dorsal hippocampus sections/animal with a focus on the CA3–CA1 region (Supplementary Fig. 3), as this hippocampal region has been shown to play an important role in the encoding and retrieval of spatial memories^99,100^. In ImageJ, three 0.1 mm^2^ boxes were drawn with the same reference position of the hippocampus proper for each section (Supplementary Fig. 3). The numbers of astrocytes (GFAP+), microglia (Iba1+), and CD206 positive microglia (Iba1+/CD206+) in each box were counted.

#### Statistics

The total number of cells in all nine boxes was calculated per mouse to provide cell density (# cells/0.9 mm^2^). The percentage of CD206 positive microglia relative to the total number of microglia cells was calculated. Two-way ANOVAs were performed in GraphPad Prism (Version 10.0.2), to determine the effect of maternal *H. bakeri* infection and offspring sex on microglial and astrocyte cell density. Values are presented as means ± SEM. The significance level was set at 0.05.

### Supplementary Information

Supplementary Figures.

Supplementary Tables.

## Data Availability

The datasets generated and analysed for the long-term potentiation, resistance and neuroimmune study are available via a link to the Borealis Dataverse [10.5683/SP3/3NQFPS], a public data repository. The dataset generated and analysed for the gene expression study was deposited in the National Center for Biotechnology Information Sequence Read Archive Database [BioProject: PRJNA1071490; https://www.ncbi.nlm.nih.gov/sra/PRJNA1071490].
