# Supplementary material for: Maternal gastrointestinal nematode infection alters hippocampal neuroimmunity, promotes synaptic plasticity, and improves resistance to direct infection in offspring
Source: Sci Rep. 2024 May 10;14:10773. doi: 10.1038/s41598-024-60865-2 (PMC11087533; doi:10.1038/s41598-024-60865-2)

Maternal gastrointestinal nematode infection alters hippocampal neuroimmunity, promotes synaptic plasticity, and improves resistance to direct infection in offspring.

Sophia C. Noel, Jeanne F. Madranges, Jean-David M. Gothié, Jessica Ewald, Austen J. Milnerwood, Timothy E. Kennedy and Marilyn E. Scott

This document contains three supplementary figures that accompany this paper.

Supplementary Figures

Supplementary Figure 1. Maternal *H. bakeri* infection did not influence dam weight at gestation day (GD) 7, 12 and 17. The identity of the dam was included as a random factor and litter size as a covariate. Values are LSmeans±SEM, n = 18-20 per group (ns = not significant).

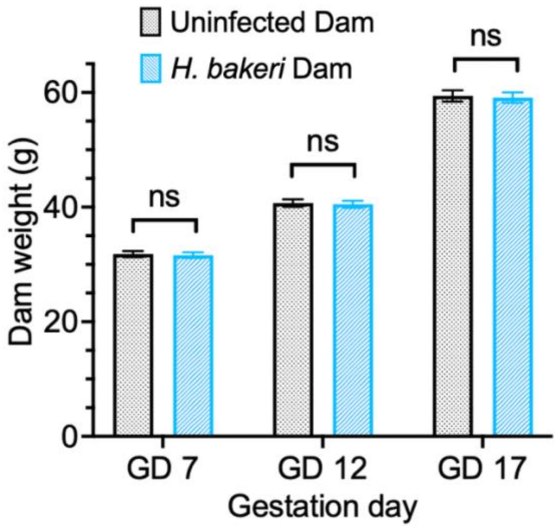

Supplementary Figure 2. Pups born to *H. bakeri* infected dams had shorter length and lower mass than pups of uninfected dams at postnatal day 20. Pups were nested within dam, and offspring sex and litter size were included as covariates. Values are LSmeans$\pm$SEM, n = 54-59 offspring per group (***P < 0.001). **(a)** pup crown-rump length and **(b)** pup body mass.

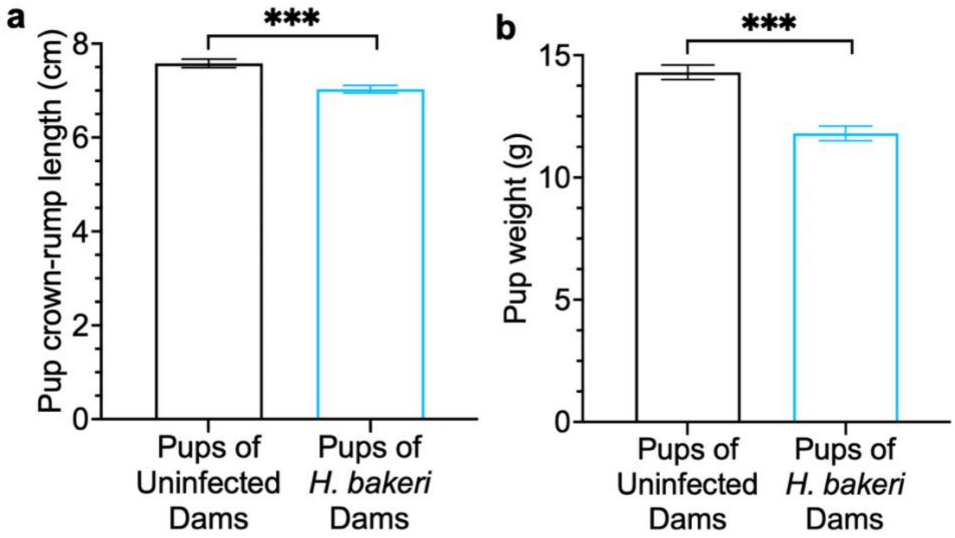

Supplementary Figure 3. Immunofluorescence labeling of dorsal hippocampus to assess density of astrocytes, microglia, and CD206 positive microglia in response to maternal *H. bakeri* infection. Astrocytes were detected by glial fibrillary acidic protein (GFAP) antibody (magenta) and microglia were detected by ionized calcium binding adapter molecule 1 (Iba1) antibody (red). To detect CD206 positive microglia, the Mouse Macrophage Mannose Receptor/CD206 antibody was used (green), and double labelled Iba1+/CD206+ cells were assessed. Cell nuclei were stained with Hoechst dye (cyan). Confocal microscope (Leica SP8) was used to image three dorsal hippocampus sections/ animal. In ImageJ, three 0.1 mm^2 boxes were drawn with the same reference position of the hippocampus proper for each section (shown in yellow). The numbers of astrocytes (GFAP+), microglia (Iba1+), and CD206 positive microglia (Iba1+/CD206+) in each box were counted and summed to provide cell density (# cells/ 0.9 mm^2/mouse). Scale bar: 300 μm.

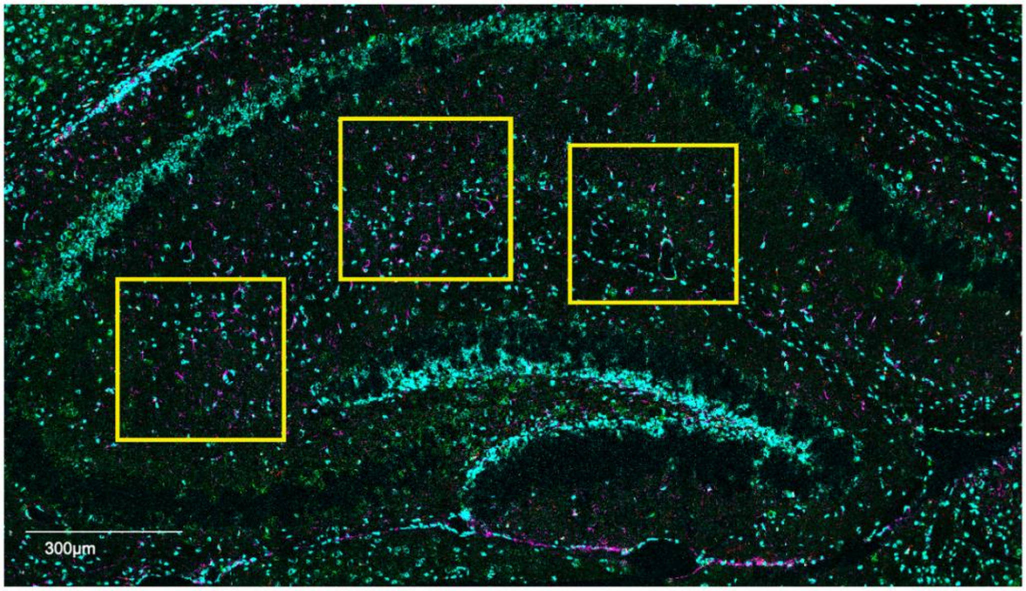

Supplement: Supplementary file 1 — Supplementary Figures. [file 41598_2024_60865_MOESM1_ESM.pdf]
